# Multimorbidity Clusters in the Oldest Old: Results from the EpiChron Cohort

**DOI:** 10.3390/ijerph191610180

**Published:** 2022-08-17

**Authors:** Ignatios Ioakeim-Skoufa, Mercedes Clerencia-Sierra, Aida Moreno-Juste, Carmen Elías de Molins Peña, Beatriz Poblador-Plou, Mercedes Aza-Pascual-Salcedo, Francisca González-Rubio, Alexandra Prados-Torres, Antonio Gimeno-Miguel

**Affiliations:** 1 WHO Collaborating Centre for Drug Statistics Methodology, Department of Drug Statistics, Division of Health Data and Digitalisation, Norwegian Institute of Public Health, NO-0213 Oslo, Norway; 2 EpiChron Research Group, Aragon Health Sciences Institute (IACS), IIS Aragón, Miguel Servet University Hospital, ES-50009 Zaragoza, Spain; 3 Drug Utilization Work Group, Spanish Society of Family and Community Medicine (semFYC), ES-08009 Barcelona, Spain; 4 Aragon Health Service (SALUD), Miguel Servet University Hospital, ES-50009 Zaragoza, Spain; 5 Research Network on Health Services in Chronic Patients (REDISSEC), ISCIII, ES-28029 Madrid, Spain; 6 Research Network on Chronicity, Primary Care and Health Promotion (RICAPPS), ISCIII, ES-28029 Madrid, Spain; 7 Primary Care Pharmacy Service Zaragoza III, Aragon Health Service (SALUD), ES-50017 Zaragoza, Spain

**Keywords:** aged, electronic health records, epidemiology, cluster, clustering, multimorbidity, multiple chronic conditions, noncommunicable diseases, public health, routinely collected health data

## Abstract

Multimorbidity is challenging for both patients and healthcare systems due to its increasing prevalence and high impact on people’s health and well-being. The risk of multimorbidity increases with age, but there is still more to discover regarding the clinical profile of the oldest old. In this study, we used information from the EpiChron Cohort Study to identify multimorbidity patterns in individuals who died during the period 2010–2019 at the ages of 80–89, 90–99, and ≥100. This cohort links the demographic, clinical, and drug dispensation information of public health system users in Aragón, Spain. We saw a significantly lower number of chronic diseases and drugs and a lower prevalence of polypharmacy in centenarians compared to those aged 80–99. K-means clustering revealed different multimorbidity clusters by sex and age group. We observed clusters of cardiovascular and metabolic diseases, obstructive pulmonary conditions, and neoplasms, amongst other profiles. One in three octogenarian women had a metabolic pattern (diabetes, dyslipidaemia, and other endocrine–metabolic disorders) with the highest number of diseases (up to seven) and prevalence of polypharmacy (64%). We observed clusters of dementia and genitourinary disorders in individuals on medication with anticholinergic activity. Our study offers an opportunity to better understand the urgency of adequately addressing multimorbidity in our older adults.

## 1. Introduction

Multimorbidity, the coexistence of two or more chronic conditions in the same individual, is an important challenge for healthcare providers and systems due to its increasing prevalence and high impact on people’s health and well-being. It is associated with negative outcomes, further morbidity, increased (and many times, inappropriate) healthcare service utilisation, a higher number of drug prescriptions (and further risks of adverse reactions, interactions, potentially inappropriate medication, and prescribing cascades), and higher costs [1,2,3]. Various initiatives have been developed and implemented for people with multiple chronic conditions [4,5,6,7,8]; however, there are uncertainties regarding the effectiveness of interventions for people with multimorbidity because of the small number of randomised clinical trials so far [9]. Therefore, there is an urgent need to advance research in the field, as the Academy of Medical Sciences (UK) highlighted [10,11]. The National Institute for Health and Care Excellence (UK) recommendations aim to identify individuals who may benefit from an approach to care that takes account of multimorbidity [12,13].

Chronic diseases tend to accumulate as we age, and there are many reasons for that. Nevertheless, the good news is that many of these reasons depend on us, or, at least, we can control some of them (together, healthcare professionals and patients) [14,15,16,17,18,19,20]. It seems that enjoying a good health status at younger old age is associated with a higher chance of enjoying healthy ageing [21,22]. In this sense, although multimorbidity is a common finding in older adults [23,24,25], a previous work showed that centenarians present, in general, a better health status than octogenarians and nonagenarians in terms of lower morbidity and treatment burden and lower use of healthcare services [26].

As some chronic diseases tend to appear together, it is crucial to study multimorbidity patterns and their clinical characteristics in depth [23,27]. Several studies have identified multimorbidity clusters in older adults [28,29,30,31,32,33]. These studies revealed the critical role of major chronic diseases in defining the clusters, especially the role of cardiovascular diseases, diabetes mellitus, respiratory diseases, neoplasms, neurologic disorders, mental health problems, and musculoskeletal and rheumatologic conditions. Although it has been reported that there are significant differences in the clinical profile of octogenarians, nonagenarians, and centenarians [34,35], only a few studies on multimorbidity clusters have focused on the population 80 years and over. The identification of multimorbidity clusters in centenarians and older adults who did not reach the age of 100 could help us detect harmful combinations of chronic diseases that should be taken into consideration in prevention programmes for the proper clinical management of chronic patients. The aim of this population-based cohort study is to identify and characterise multimorbidity clusters in octogenarians, nonagenarians, and centenarians and describe their morbidity burden.

## 2. Materials and Methods

We conducted a retrospective, observational study in the EpiChron Cohort [25]. This cohort includes clinical and demographic data from public health system users in Aragón (Spain) by linking information from electronic health records and clinical-administrative databases. The full profile of the EpiChron Cohort, along with key findings regarding the baseline clinical characteristics of the included population (approximately 1.3 M individuals as of 1 January 2011, corresponding to 98% of the total inhabitants in the region), has been published elsewhere [25]. All of the information registered in the EpiChron Cohort is anonymised, and it includes data from primary care, hospital care, specialist care, emergency rooms, pharmaceutical billing, and the Patient Index Database.

In this study, we included all individuals from the EpiChron Cohort with at least one contact with the health system registered in their electronic health records and who died at an oldest-old age (80 years or older) during the period 1 January 2010 to 31 December 2019. We further stratified the study population by sex and age-at-death group (octogenarians, 80–89; nonagenarians, 90–99 years; and centenarians, ≥100 years). To identify differences in the clinical profile between centenarians and other oldest old who were not able to reach the age of 100, we only studied deceased individuals, as in previous works [26].

For each individual, we included information regarding sex, age, chronic disease diagnoses registered in primary care and/or hospital electronic health records, medications dispensed, presence of polypharmacy (≥5 drugs), excessive polypharmacy (≥10 chronic drugs), and Anticholinergic Drug Scale (ADS) score [36,37,38]. Age and drug use information corresponded to the last 365 days of follow-up for each individual. Chronic diseases were included if their status in the patient’s clinical history was “active” during the 365-days-prior-to-death follow-up period; they can be diagnosed and registered throughout the patient’s lifetime.

Primary care diagnoses registered according to the first edition of the International Classification of Primary Care (ICPC-1) were mapped to the ninth edition, clinical modification of the International Classification of Diseases (ICD-9-CM) [39]. Specialised software was used to first sort all diagnoses into 226 clinically relevant categories (Clinical Classifications Software, CCS) [40] and to then classify them into chronic and nonchronic conditions (Chronic Condition Indicator software tool) [41]. Finally, 153 conditions were categorised as chronic. Some of the diagnostic labels were renamed or grouped together to facilitate their clinical interpretation. We analysed all drugs using the World Health Organization’s Anatomical Therapeutic Chemical (ATC) classification system [42]; we used the third ATC level (i.e., pharmacological subgroup) to study the number of drugs and the fifth ATC level (i.e., substance) to calculate the anticholinergic score.

We applied descriptive statistics to demographic and clinical information for each age group, stratified by sex. For comparisons between age groups we used the ANOVA test applying the Bonferroni multiple-comparison correction for means and the Pearson’s chi-squared test for frequencies. For comparisons within each age group, the Student’s *t*-test and Pearson’s chi-squared test were used, respectively.

We applied cluster analysis to identify clusters of similar individuals according to their chronic conditions in each age and sex group, as previously described [43,44]. A total of 91,442 patients were included in the cluster analysis. Only chronic conditions with a prevalence of ≥1% were included in the analysis. Each chronic condition was considered a dichotomous variable (presence/absence).

As described in a previous study, we followed an optimisation process for the selection of the clustering method [44]. An agglomerative hierarchical method was first run, but we finally performed a k-means non-hierarchical analysis because of computational limitations. To establish the distance between individuals, we used the Jaccard coefficient as a measure of similarity. To properly select the most optimum number of clusters for each age and sex group, we started from the lowest number of clusters that maximised intra-group homogeneity and inter-group heterogeneity, offering a consistent clinical interpretation; the Calinski and Harabasz statistic was also considered [45]. The selection of the number of clusters and their clinical interpretation followed a process in which the clinical partners (I.I.-S., M.C.-S., A.M.-J., C.E.d.M.P., F.G.-R., and A.P-T.) discussed it in consecutive rounds until a consensus was reached. Then, we calculated the prevalence of every single disease in each multimorbidity cluster. For every single condition, we expressed differences between the observed prevalence (OP) in each multimorbidity cluster and the expected prevalence (EP; i.e., the prevalence of the chronic condition in the corresponding sex and age group) as prevalence ratios (PR; PR = OP/EP). Both the prevalence of every single disease in a cluster and its PR were taken into consideration in defining the particular clinical profile (disease composition) of each cluster. A disease was considered an essential part of the clinical profile of a multimorbidity cluster if: (i) its prevalence was higher than 10% and PR was >2, or (ii) its prevalence was higher than 20% and PR was >1.2.

Clusters were compared using Pearson’s chi-squared test for categorical variables, ANOVA to compare means, and the Kruskal–Wallis test when the data were not normally distributed. All analyses were conducted in STATA software (Version 12.0, StataCorp LLC, College Station, TX, USA), with the statistical significance set at *p* < 0.05.

## 3. Results

### 3.1. Demographic and Clinical Characteristics of the Study Population

A total of 91,442 individuals (56.1% women) of the EpiChron Cohort died at age 80 or older from 2011 to 2019. Centenarians represented only 2.5% of the study population, and approximately 80% were women. Table 1 summarises demographic and clinical information on the study population. The mean number of chronic conditions and drugs was lower in centenarians compared with younger older adults: from approximately six chronic diseases and five medications in octogenarians to less than five conditions and less than four drugs in centenarians. The prevalence of polypharmacy, especially excessive polypharmacy, was significantly lower in centenarians than in other age groups. Almost half of the study population was dispensed at least one medication with some anticholinergic activity; however, centenarians showed a significantly lower use of such medications.

Hypertension was the most prevalent chronic condition in the study population. Amongst the most common chronic diseases were genitourinary disorders, visual deficits, chronic skin ulcers, osteoarthritis, delirium and dementia, ictus, cardiac arrhythmias, and congestive heart failure. Table S1 in the Supplementary Material provides an overview of the twenty most prevalent conditions by age group and gender. The prevalence of genitourinary disorders was higher in centenarians compared with octogenarians. On the other hand, the prevalence of diabetes mellitus and lipid metabolism disorders was substantially lower with advanced age.

K-means clustering revealed different multimorbidity clusters by age group and gender. Below is a brief description of the clusters highlighting their clinical characteristics; see Table 2 and Table 3. We observed significant differences in the morbidity burden (number of chronic diseases and drugs, prevalence of polypharmacy, and use of medication with anticholinergic activity) between the identified clusters. The complete clinical description of the multimorbidity clusters is given in Table S2, and the *p*-values of the differences between them are provided in Table S3, both in Supplementary Material.

### 3.2. Multimorbidity Clusters in Octogenarians

In octogenarian men, we identified three multimorbidity clusters. Hypertension, dyslipidaemia, diabetes mellitus, other endocrine–metabolic diseases, cardiovascular conditions (such as cardiac arrhythmias and non-hypertensive congestive heart failure), and chronic kidney disease were systematically associated in the most prevalent multimorbidity cluster. This cluster had the highest morbidity burden, with an average of approximately six chronic diseases and more than five drugs. More than half of the individuals in this cluster had polypharmacy, and one in two had excessive polypharmacy. Almost half of the individuals in this cluster had at least one medication with some anticholinergic activity. However, the cluster with the highest anticholinergic score in octogenarian men was the one with genitourinary disorders, delirium and dementia, ictus, and chronic skin ulcers. Almost three in ten octogenarian men presented this profile, with an average of approximately five to six diseases and up to five drugs. The cluster with the lowest burden of chronic diseases and medications (an average of three conditions and three drugs) was the cluster with a high prevalence of neoplasms and chronic obstructive pulmonary disease, present in one in every ten octogenarian men. This cluster had the lowest prevalence of polypharmacy and the lowest anticholinergic score.

In octogenarian women, we identified four multimorbidity clusters. Two of them were the most prevalent ones (60% together)—endocrine–metabolic and cardiovascular. One in three women had a metabolic pattern of dyslipidaemia, diabetes mellitus, or other endocrine–metabolic conditions; this cluster was associated with the highest number of chronic diseases and drugs, excessive polypharmacy (one in four women), and the highest anticholinergic score in the whole study population (score of ≥3 in 23.7% and ≥5 in 8% of the individuals). The cardiovascular cluster included individuals with cardiac arrhythmias, non-hypertensive congestive heart failure, and coagulation and haemorrhagic disorders. Hypertension was highly prevalent in both clusters (endocrine–metabolic and cardiovascular). However, there was no dyslipidaemia or other endocrine–metabolic conditions in the cardiovascular cluster. We identified two more multimorbidity clusters in octogenarian women, a cluster of osteoarthritis (present in one in five women) and a cluster of delirium and dementia. In this last cluster, eight in ten women had delirium/dementia, and four in ten women had been on medication with some anticholinergic activity.

### 3.3. Multimorbidity Clusters in Nonagenarians

In nonagenarian men, we identified four multimorbidity clusters. Three in four individuals were grouped into two different clusters of cardiometabolic conditions. The cardiovascular cluster, present in three in ten men, included cardiac arrhythmias, non-hypertensive heart failure, and a high prevalence of other comorbidities such as chronic kidney disease and coagulation and haemorrhagic disorders. It had the highest morbidity burden, with more than six chronic conditions, six drugs, increased use of medication with anticholinergic activity, and excessive polypharmacy in one in five individuals. The endocrine–metabolic cluster included individuals with hypertension, dyslipidaemia, and diabetes mellitus, but not cardiac arrhythmias. We identified two more multimorbidity clusters in nonagenarian men: one with genitourinary disorders, delirium and dementia, ictus, and chronic skin ulcers, and one with a high prevalence of neoplasms, chronic obstructive pulmonary disease, visual deficits, and prostate hyperplasia and characterised by the lowest number of chronic conditions and drugs, the lowest prevalence of polypharmacy, and the lowest anticholinergic score.

In nonagenarian women, we identified five multimorbidity clusters. One in three individuals was included in a cluster of delirium and dementia, with the highest number of comorbidities and the highest anticholinergic score in this age group. Another high-prevalence cluster included women with hypertension; this cluster had no ictus. Individuals with ictus and without delirium/dementia were grouped in a different cluster, with approximately six chronic conditions and five drugs. We also identified a less prevalent cluster of chronic skin ulcers and a cluster of genitourinary disorders and non-hypertensive congestive heart failure.

### 3.4. Multimorbidity Clusters in Centenarians

In centenarian men, we identified three multimorbidity clusters. A cluster of genitourinary disorders and chronic skin ulcers included almost half of the individuals in this group. Another cluster included men with acute myocardial infarction, thyroid disorders, delirium and dementia, and other conditions but not hypertension. This cluster had the lowest morbidity burden (an average of approximately three simultaneous chronic conditions). Hypertension was highly prevalent in a multimorbidity cluster with various chronic conditions, such as anaemias, ictus, visual deficits, and osteoarthritis; approximately 70% of the individuals in this cluster had no drugs with anticholinergic activity, and less than 5% had excessive polypharmacy. We found no statistically significant differences regarding medications (such as the number of drugs, polypharmacy, and anticholinergic medication) between multimorbidity clusters in centenarian men.

In centenarian women, we identified five multimorbidity clusters. One in four individuals was included in a cluster of chronic ulcers of the skin with an average of four additional comorbidities. One in ten women was in a cluster of endocrine–metabolic disorders, dyslipidaemia, diabetes, delirium/dementia, and hypertension. However, hypertension was mainly diagnosed amongst individuals of another cluster, the most prevalent one in centenarian women (four in ten individuals), with osteoarthritis, visual deficits, and other comorbidities. Two other multimorbidity clusters were also identified: one with a high prevalence of congestive heart failure and with an average of one chronic condition and less than two drugs, and the other one with genitourinary disorders and ictus; neither of these two clusters had hypertension.

## 4. Discussion

Multimorbidity clusters in the oldest old, as well as their morbidity burden, vary substantially between specific age groups and by gender. An in-depth study of conditions whose coexistence determines the particularities of each cluster could help in the future to identify clinically similar groups of patients who may benefit from similar approaches to care that take account of specific multimorbidity profiles.

In our study, only a few of the oldest old died as centenarians (approximately one in forty individuals), most of whom were women. Similar findings have been widely reported in the literature [34,46,47,48,49,50,51,52,53,54,55], and various factors have been hypothesised to explain these observations [15,56]. It has been reported that geriatric syndromes are more common in women than in men, and this could be due to various reasons, including poorer treatment adherence and potential sex differences in functional reserve [49,57]. It seems that men are, in general, healthier than women, but they die younger; this is the “health–survival paradox” [57], and numerous biological, behavioural, and social factors may explain it [57,58,59]. A common example of behavioural factors is that men tend to ignore symptoms, feeling healthier than they actually are, and do not seek medical care as frequently as women do [58]. Individuals at high risk of frailty and severe conditions that could significantly compromise their health status may drop out of the population before reaching the age of 100 [50,60,61]. Centenarians had, in general, a lower mean number of chronic conditions and drugs, as well as a lower prevalence of excessive polypharmacy, in comparison with the oldest old who died at a younger age. Individuals who achieved longevity seemed to enjoy relatively good health and functional status at younger ages [35,49,60,62].

Hypertension, genitourinary disorders, visual deficits, chronic skin ulcers, delirium and dementia, osteoarthritis, and ictus were amongst the most common chronic conditions in all age groups of the study population. We saw that genitourinary disorders and chronic skin ulcers were more prevalent in the population over 100. However, in general, most chronic conditions had a lower prevalence in centenarians than in the youngest oldest old. Examples were dyslipidaemia and diabetes mellitus, with an approximately three times lower prevalence in centenarians than in octogenarians. Other common conditions less prevalent in centenarians were neoplasms and acute myocardial infarction. Other authors have documented similar findings, suggesting that conditions with high mortality risk are more prevalent in younger age groups [34,50]. A study showed that, at the same age, those living longer had a lower prevalence of most chronic diseases than those who died at an earlier age; the authors reported remarkable differences regarding dementia and chronic heart disease [63].

The existence of systematic associations between diseases is well known; chronic diseases tend to cluster, non-randomly, in groups commonly called multimorbidity clusters or multimorbidity patterns [27,64,65]. The identification of these associations beyond chance is essential to the in-depth study of the underlying biological interactions amongst diseases and their clinical implications [27,28,32,64,65]. Various statistical techniques have been used to study these clusters in a variety of clinical settings and population groups, from simple combinations of diseases (dyads or triads) to meaningful patterns (by factor analysis, cluster analysis, network analysis, and so forth) [28,32,44,66,67]. For example, it has been found that visual impairment and cardiovascular diseases (such as heart failure) were comorbidities among the oldest old population’s most prevalent pairs of chronic diseases [28]. In this study, we applied cluster analysis to identify clusters of individuals based on their clinical profiles. Although the consistency of the identified clusters varied by sex and age group, we saw that some diseases and combinations could determine the particularities of various clusters in different age groups or both sexes. Such combinations include dyslipidaemia and diabetes mellitus, delirium or dementia and chronic skin ulcers, cardiac arrhythmias and congestive heart failure, and neoplasms and chronic obstructive pulmonary disease. In some cases, the clinical profile of the multimorbidity clusters includes combinations of diseases that are not necessarily in the same body system (or clinically managed by the same medical specialty), and sometimes their relationship is not clinically well documented. Using robust statistical techniques, large-scale epidemiologic studies can reveal unexpected associations with high relevance to clinical practice [27].

As a good health status at younger old age is associated with better chances for longevity [49,60,62], it is vital to perform in-depth studies on the clinical profiles of the oldest old, especially for individuals who died as octogenarians or nonagenarians. In older adults, multimorbidity is the rule rather than the exception [23,24,25]. It is logical to think that some combinations of diseases may have a higher impact on health than others and that pharmacological management plays a vital role in clinical outcomes. The high mean number of diseases and drugs in older adults may involve disease–disease, drug–drug, and drug–disease interactions, as well as prescribing cascades [68,69], increasing the risk of potentially inappropriate medication and its clinical consequences [70,71,72]. An interesting finding, for example, was the high use of drugs with anticholinergic activity in clusters with dementia, especially in octogenarians and nonagenarians. Anticholinergic medication, sometimes very common amongst antidepressants and urologicals, is a potentially modifiable risk factor for dementia and cognitive impairment [73,74,75].

Our study revealed that one in three women who died as octogenarians had dyslipidaemia and a particularly high prevalence of other metabolic conditions. It included almost all women with other nutritional–endocrine disorders (three in five individuals in the cluster) and many with diabetes mellitus and hypertension. Moreover, more than one in five women in this cluster had been prescribed at least ten drugs during their last year of life. This cluster had the highest mean number of diseases and drugs and the highest anticholinergic score in the whole study population. In octogenarian men, cardiovascular comorbidity was included in the same cluster as metabolic diseases. In a previous study, we found that metabolic conditions were systematically associated and that this pattern was present even in young adults [32]. Individuals in this cluster may be at a higher risk of metabolic syndrome and cardiovascular comorbidity [31,32,76]. Although this cluster was the one with the highest morbidity burden in the oldest old, it is essential to highlight that many of the significant conditions of this profile were preventable (for example, dyslipidaemia and diabetes mellitus).

Notably, inflammation is a common factor in many chronic diseases that play a central role in most multimorbidity clusters. We saw cardiovascular diseases, neoplasms, diabetes, ictus, chronic obstructive pulmonary disease, and dementia; although these conditions seem clinically different, there is evidence that they share a common inflammatory background [15,77,78]. Although there is still much to learn, more and more studies are underlining the physiological mechanisms of systemic low-grade chronic inflammation and its impact on health [14,15,16,17,19,77,79,80]. In the elderly, the age-related chronic state of low-grade systemic inflammation, commonly known as inflammageing, is implicated in the pathogenesis of numerous chronic inflammatory diseases, and it is a risk factor for mortality [15,77,79,81,82]. Inflammageing results from many factors, including genetics, ageing, and lifestyle [14,15,16,17,18,19]. Adopting healthy lifestyle factors could prevent the development of chronic inflammation and certain chronic diseases. Sometimes, it requires much effort, as lifestyle is the way we live, so it can be hard to change it; special reports have been recently published to inform the general public on how to prevent or fight chronic inflammation, such as the one from Harvard Medical School [20].

One of the main strengths of this cohort study is its population-based nature. Moreover, we exhaustively analysed the multimorbidity clusters’ clinical profiles using information from primary and hospital care electronic health records, including all chronic conditions and not only the most common or severe diseases. Data in the EpiChron Cohort continuously undergo quality control check-ups that ensure its accuracy and reliability for research. A significant limitation of the study is that we did not consider the time of onset of the diseases nor the chronological appearance of additional comorbidities over time. Another limitation was that we had no data regarding biochemical and genetic information, lifestyle, and other socio-demographic factors of great interest. Further research is necessary to study multimorbidity clusters’ development and clinical evolution in the oldest old.

## 5. Conclusions

This study revealed that the clinical profile of multimorbidity clusters in older adults varies substantially by sex and age group. In octogenarians, we saw clusters with a particularly high morbidity burden and a high prevalence of preventable chronic conditions. Our results highlight the urgency of implementing effective public health strategies to adequately prevent the development of specific chronic conditions, such as dyslipidaemia and diabetes mellitus, even in younger adults. A person-centred and integrated care model is essential for the proper clinical management of the oldest old in all clinical settings, especially in primary care. A comprehensive assessment of the clinical profile, functional status, mental health, and socio-economic status, considering patient preferences, is key to ensuring the optimal clinical management of our older adults.

## Figures and Tables

**Table 1:** Demographic and clinical characteristics of the study population by age group (dead at the age of 80–89, octogenarians; 90–99, nonagenarians; or ≥100 years, centenarians) and sex (M, men; W, women).

Demographic and Clinical Information ^a^	Octogenarians			Nonagenarians			Centenarians			*p*_men_ ^b^			*p*_women_ ^b^		
	M	W	*p*	M	W	*p*	M	W	*p*	*p*_octo-nona_	*p*_nona-cent_	*p*_octo-cent_	*p*_octo-nona_	*p*_nona-cent_	*p*_octo-cent_
Total Population (n)	26,758	26,563		12,929	22,896		466	1 830							
Age (mean)	84.70	85.25	0.000	92.81	93.35	0.000	101.62	101.62	0.956	0.000	0.000	0.000	0.000	0.000	0.000
Number of Chronic Diseases (mean)	5.84	6.23	0.000	5.57	5.70	0.000	4.36	4.51	0.229	0.000	0.000	0.000	0.000	0.000	0.000
Number of Drugs (mean)	5.03	5.09	0.167	4.95	4.77	<0.001	3.47	3.58	0.563	0.270	0.000	0.000	0.000	0.000	0.000
Polypharmacy ^c^ (%)	52.71	52.91	0.640	53.72	52.31	0.01	36.48	38.20	0.495	ns	<0.001	<0.001	<0.001	<0.001	ns
Excessive Polypharmacy ^d^ (%)	18.94	20.07	0.001	15.66	14.29	0.000	6.87	6.50	0.777	<0.001	<0.001	<0.001	<0.001	<0.001	<0.001
ADS ^e^ (mean)	1.21	1.25	0.002	1.19	1.21	0.517	1.04	0.95	0.320	1.000	0.161	0.119	0.017	0.000	0.000
ADS ^e^ score (%)			0.001			0.026			0.331	0.015	0.015	0.015	0.000	0.000	0.000
0	54.57	52.65		53.72	52.43		60.52	59.95		ns	<0.001	ns	ns	<0.001	ns
1	15.37	15.96		15.89	16.66		13.73	15.74		ns	ns	ns	ns	ns	<0.001
2	10.43	10.96		10.56	11.48		8.15	9.62		ns	ns	ns	ns	<0.001	<0.001
3	7.92	8.34		8.59	8.35		7.51	6.39		<0.001	ns	<0.001	ns	<0.001	ns
4	4.94	5.22		5.13	5.03		4.29	4.43		ns	ns	ns	ns	ns	ns
≥5	6.78	6.87		6.10	6.05		5.79	3.88		<0.001	ns	<0.001	<0.001	<0.001	<0.001

^a^ Multiple-comparison. For means: ANOVA, Bonferroni test; for frequencies (%): Pearson’s chi-squared test, adjusted residual (associated categories if Adj. res > |1.96|). ^b^ *p*_men_ and *p*_women_ represent the *p*-values of comparisons among men of different age groups and women of different age groups, respectively. *p*_octo-nona_ represents the *p*-values of comparisons between octogenarians and nonagenarians, *p*_octo-cent_ represents the *p*-values of comparisons between octogenarians and centenarians, and *p*_nona-cent_ represents the *p*-values of the comparisons between nonagenarians and centenarians. ns, not significant, adj. residual < |1.96|. ^c^ Five or more medications. ^d^ Ten or more medications. ^e^ ADS, Anticholinergic Drug Scale.

**Table 2:** Multimorbidity clusters in oldest old men by age group (octogenarians, MO1, MO2, and MO3; nonagenarians, MN1, MN2, and MN3, and MN4; centenarians, MC1, MC2, and MC3).

Cluster	Prev ^a^ (%)	Number of Conditions (Mean)	Conditions ^b^	Number of Drugs (Mean)	PP ^c^ (%)	EPP ^d^ (%)	ADS ^e^ Mean Score	ADS Score ^f^ (%)					
								0	1	2	3	4	≥5
**Octogenarians**													
MO1	57.92	6.1	HT, dyslipidaemia, DM, cardiac arrhythmias, endocrine–metabolic disorders, coagulation/haemorrhagic disorders, CHF, KD.	5.5	57.1	22.2	1.2	53.4	17.2	10.1	7.9	5.0	6.6
MO2	31.97	5.6	Genitourinary symptoms, delirium and dementia, ictus, chronic ulcer of skin.	4.8	51.2	16.3	1.3	54.0	13.1	11.9	8.6	5.1	7.4
MO3	10.11	3.1	COPD, neoplasms.	3.1	32.1	8.3	1.0	63.2	12.5	8.0	6.1	4.1	6.1
**Nonagenarians**													
MN1	43.81	5.5	HT, dyslipidaemia, DM.	5.2	56.6	16.5	1.2	53.2	16.8	10.0	9.0	5.3	5.8
MN2	30.20	6.7	Cardiac arrhythmias, coagulation/haemorrhagic disorders, CHF, KD.	5.9	64.3	21.2	1.4	48.0	18.1	11.9	8.7	5.9	7.4
MN3	18.09	4.1	Genitourinary symptoms, delirium and dementia, ictus, chronic ulcer of skin.	3.7	39.5	8.7	1.1	58.7	12.1	11.0	8.3	4.3	5.6
MN4	7.90	2.5	COPD, neoplasms, visual deficits, prostate hyperplasia.	2.8	30.0	5.9	0.8	67.2	11.3	7.4	6.7	3.2	4.2
**Centenarians**													
MC1	51.29	4.6	Genitourinary symptoms, chronic ulcer of skin.	3.7	38.5	7.5	1.1	55.2	16.3	9.6	7.5	5.4	5.9
MC2	35.84	3.9	HT, visual deficits, ictus, osteoarthritis, anaemias.	3.1	33.5	4.8	0.9	69.5	9.0	4.8	8.4	2.4	6.0
MC3	12.88	2.9	CHF, delirium and dementia, cardiac arrhythmias, COPD, acute myocardial infarction, thyroid disorders.	3.4	36.7	10.0	1.1	56.7	16.7	11.7	5.0	5.0	5.0

^a^ Prev, prevalence of each cluster in the corresponding sex and age group. ^b^ Chronic conditions. Some abbreviations: HT, hypertension, DM diabetes mellitus, CHF, non-hypertensive congestive heart failure; KD, chronic kidney disease; COPD, chronic obstructive pulmonary disease. ^c^ PP, prevalence of polypharmacy defined as five or more medications. ^d^ EPP, prevalence of excessive polypharmacy defined as ten or more medications. ^e^ ADS, Anticholinergic Drug Scale given as mean score. ^f^ ADS score, Anticholinergic Drug Scale score given as prevalence of individuals with scores of 0, 1, 2, 3, 4, and 5 or higher.

**Table 3:** Multimorbidity clusters in oldest old women by age group (octogenarians, WO1, WO2, WO3, and WO4; nonagenarians, WN1, WN2, WN3, WN4, and WN5; centenarians, WC1, WC2, WC3, WC4, and WC5).

Cluster	Prev ^a^ (%)	Number of Conditions (Mean)	Conditions ^b^	Number of Drugs (Mean)	PP ^c^ (%)	EPP ^d^ (%)	ADS ^e^ Mean Score	ADS Score ^f^ (%)					
								0	1	2	3	4	≥5
**Octogenarians**													
WO1	36.72	7.7	Dyslipidaemia, DM, endocrine–metabolic disorders.	6.3	64.3	27.3	1.5	45.0	18.6	12.7	9.5	6.1	8.0
WO2	22.75	4.6	Cardiac arrhythmias, CHF, coagulation/haemorrhagic disorders.	4.4	45.9	16.6	1.0	59.5	14.9	9.2	6.7	4.1	5.6
WO3	21.89	6.0	Osteoarthritis.	4.9	51.3	18.7	1.3	52.6	16.4	10.6	8.0	5.3	7.2
WO4	18.65	4.7	Delirium and dementia, chronic ulcer of skin.	3.8	40.9	11.7	1.1	59.3	11.6	10.2	8.5	4.7	5.7
**Nonagenarians**													
WN1	34.09	6.2	Delirium and dementia.	4.7	52.1	13.0	1.3	49.5	14.7	13.9	10.0	5.1	6.8
WN2	32.17	5.1	Hypertension.	4.9	53.7	15.3	1.2	53.2	18.1	10.3	7.6	5.3	5.4
WN3	17.06	5.9	Ictus.	5.1	55.7	16.5	1.2	53.0	18.1	10.4	7.5	5.3	5.9
WN4	10.16	5.7	Chronic ulcer of skin.	4.9	53.3	15.8	1.3	51.3	17.9	10.4	8.5	4.9	7.1
WN5	6.52	3.1	Genitourinary symptoms, CHF.	3.3	35.8	8.0	0.9	64.1	14.0	9.4	5.5	3.1	3.9
**Centenarians**													
WC1	41.04	4.3	HT, osteoarthritis, visual deficits.	4.0	42.9	7.2	1.0	56.3	17.3	11.9	5.9	5.2	3.5
WC2	26.61	4.9	Chronic ulcer of skin.	3.6	38.0	7.2	1.0	60.0	14.6	8.4	7.6	4.7	4.7
WC3	15.19	3.2	Genitourinary symptoms, ictus.	3.0	31.3	4.0	0.9	62.2	16.6	7.6	6.8	3.6	3.2
WC4	11.64	5.3	Delirium and dementia, dyslipidaemia, endocrine–metabolic disorders.	3.8	43.2	8.0	1.1	59.6	14.1	10.8	6.1	3.8	5.6
WC5	5.52	1.0	CHF.	1.3	12.9	2.0	0.4	81.2	10.9	2.0	4.0	1.0	1.0

^a^ Prev, prevalence of each cluster in the corresponding sex and age group. ^b^ Chronic conditions. Some abbreviations: HT, hypertension; DM, diabetes mellitus, CHF, non-hypertensive congestive heart failure; KD, chronic kidney disease; COPD, chronic obstructive pulmonary disease. ^c^ PP, prevalence of polypharmacy defined as five or more medications. ^d^ EPP, prevalence of excessive polypharmacy defined as ten or more medications. ^e^ ADS, Anticholinergic Drug Scale given as mean score. ^f^ ADS score, Anticholinergic Drug Scale score given as prevalence of individuals with scores of 0, 1, 2, 3, 4, and 5 or higher.

## Data Availability

The data used in this study cannot be publicly shared because of restrictions imposed by the Aragon Health Sciences Institute (IACS) and asserted by the Clinical Research Ethics Committee of Aragon (CEICA, ceica@aragon.es). The authors who accessed the data belong to the EpiChron Research Group of IACS and received permission from IACS to utilise the data for this specific study, thus implying its exclusive use by the researchers appearing in the project protocol approved by CEICA. Potential collaborations should be addressed to the Principal Investigator of the EpiChron Research Group, Alexandra Prados-Torres, at sprados.iacs@aragon.es.

## Supplementary Materials

The following supporting information can be downloaded at: https://www.mdpi.com/article/10.3390/ijerph191610180/s1, Table S1: The twenty most prevalent chronic conditions in the study population by age group and sex, Table S2: Clinical composition (prevalence of chronic conditions) in each multimorbidity cluster found in the study population, Table S3: Statistical significance (*p*-values) of the differences between multimorbidity clusters by age group and sex.
